# A large temperature fluctuation may trigger an epidemic erythromelalgia outbreak in China

**DOI:** 10.1038/srep09525

**Published:** 2015-03-30

**Authors:** Tao Liu, Yonghui Zhang, Hualiang Lin, Xiaojuan Lv, Jianpeng Xiao, Weilin Zeng, Yuzhou Gu, Shannon Rutherford, Shilu Tong, Wenjun Ma

**Affiliations:** 1 Guangdong Provincial Institute of Public Health, Guangdong Provincial Center for Disease Control and Prevention, Guangzhou, 511430, China; 2 Environment and Health, Guangdong Provincial Key Medical Discipline of Twelfth Five-Year Plan, Guangzhou, 511430, China; 3 Guangdong Provincial Center for Disease Control and Prevention, Guangzhou, 511430, China; 4 Southern Medical University, Guangzhou, 510515, China; 5 Jinan University, Guangzhou, 510632, China; 6 Centre for Environment and Population Health, Griffith University, Brisbane, 4111, Australia; 7 Queensland University of Technology, Brisbane, Australia

## Abstract

Although erythromelalgia (EM) has been documented in the literature for almost 150 years, it is still poorly understood. To overcome this limitation, we examined the spatial distribution of epidemic EM, and explored the association between temperature fluctuation and epidemic EM outbreaks in China. We searched all peer-reviewed literature on primary epidemic EM outbreaks in China. A two-stage model was used to characterize the relationship between temperature fluctuation and epidemic EM outbreaks. We observed that epidemic EM outbreaks were reported from 13 provinces during 1960–2014 and they mainly occurred between February and March in southern China. The majority of EM cases were middle school students, with a higher incidence rate in female and resident students. The major clinical characteristics of EM cases included burning, sharp, tingling and/or stinging pain in toes, soles and/or dorsum of feet, fever, erythema and swelling. A large “V”-shaped fluctuation of daily average temperature (TM) observed during the epidemic EM outbreaks was significantly associated with the number of daily EM cases (β = 1.22, 95%CI: 0.66 ~ 1.79), which indicated that this “V”-shaped fluctuation of TM probably triggered the epidemic EM outbreaks.

Erythromelalgia (EM) is characterized by a clinical syndrome of erythema, fever, and associated discomfort, including burning pain, tingling, or similar sensations, preferentially involving the extremities brought on or aggravated by standing, walking or heat and relieved by the horizontal position and by cold^1^,^2^. EM was named by Weir Mitchell in 1878 based on his research results^2^. The first case of what became later known as EM was reported by the Irish physician Robert James Graves in 1834^3^. Although EM has been documented in the literature for almost 150 years^2^, it is still poorly understood. A few recent studies demonstrated that EM patients had lower quality of life, higher risks of disability, mortality and morbidity^1^,^4^. For instance, Friberg et al. investigated the quality of life (QOL) among EM patients in New Zealand, and found that they had significantly worse scores in the categories of physical function, bodily pain, general health, vitality, social function, emotional roles, and mental health compared with healthy respondents^1^. Some severe EM patients have reported to systematically cool their feet in iced water for up to 20 hours a day to relieve the burning pain in their feet^5^.

EM occurs either as a primary or secondary disorder^5^,^6^. Secondary EM occurs in association with various conditions including small fibre peripheral neuropathy of any cause such as diabetes, or secondary to myeloproliferative diseases, mushroom poisoning, as a side effect of some medications or as a paraneoplastic syndrome^4^,^7^,^8^,^9^,^10^. Primary EM has no identifiable causes. A number of hypotheses have been proposed including vascular shunting^11^, neuropathic aetiologies^12^, microvascular aetiologies^13^, and inflammatory aetiologies^14^. Some researchers have found that SCN9A gene mutations might be associated with primary EM^15^.

To date, few studies have investigated the prevalence of EM in western countries. A study conducted in Norway estimated that the annual incidence was 2/100,000^5^. In a population-based study of EM in the USA, the reported annual incidence was 1.3/100,000^16^, and the overall annual population-based incidence of EM in Sweden was 0.36/100,000^17^. Although data on the prevalence of EM are very limited, it is proposed that EM may be more common than previously thought^5^. In China, Zhu reported the first clinical EM case in 1945^18^ and the first epidemic EM event in 1960^19^. Since then, at least 100 studies have reported more than 80,000 EM cases in China, of which most cases were primary epidemic EM patients^20^,^21^,^22^,^23^,^24^,^25^,^26^,^27^,^28^,^29^,^30^,^31^,^32^,^33^,^34^,^35^,^36^,^37^. For example, during February and March of 1990, a total of 11,589 epidemic EM cases were reported in 13 cities/counties of Hainan province and most of these cases were in middle school students^24^. In early 1987, 219 middle schools across 29 cities/counties of Hubei province reported a total of 19,278 epidemic EM cases and most of them were female students^38^. Between late February and early March of 2014, another epidemic EM outbreak with 494 cases was reported from two middle schools of Foshan city, Guangdong province, southern China. These reports suggest that epidemic EM is common in China, but the importance of this epidemic condition has not fully been recognized by international researchers due to language barrier^39^.

The etiology of epidemic EM outbreaks in China remains unclear. Some previous studies conducted in middle school students reported possible risk factors including heavy academic burden, poor living conditions and nutrition supply, menarche, poxviruses and climatic factors^28^,^39^,^40^. Some studies found that an epidemic EM outbreak often followed a large ambient temperature fluctuation between February and March hypothesizing that the sharp drop and subsequent rapid rising of ambient temperature possibly induced the dysfunction of peripheral vasodilation and hence induced the EM^28^,^29^,^38^. However, until now this hypothesized relationship between temperature and epidemic EM has not been tested quantitatively, and some other questions about choice of temperature indicator (average temperature, maximum temperature, minimum temperature, or their corresponding apparent temperature) to best predict an EM outbreak have remained unanswered. Answering these questions will extend our understanding of the mechanisms of epidemic EM, and provide important information for the control and prevention of this disease in the future.

In order to fill this knowledge gap, we searched all published papers from various databases that described epidemic EM in China, and conducted a comprehensive analysis using data extracted from these papers. We aimed to understand the spatial distribution of epidemic EM, and explore the association between temperature fluctuation and epidemic EM outbreaks in China.

## Methods

### Literature search

We searched all literature published between January 1^st^, 1956 and March 31^st^ 2014 and indexed in MEDLINE, PubMed, China National Knowledge Infrastructure (CNKI), Wanfang, SinoMed and Chong Qing VIP (CQVIP) databases. The database searches used the following keywords: “erythromelalgia”, “erythermalgia”, “erythema”, “burning pain”, “increased temperature”, or “extremity”. We also manually searched other relevant references listed in published papers. Only peer-reviewed publications in English or Chinese were considered.

### Inclusion and exclusion criteria

**Figure 1:**
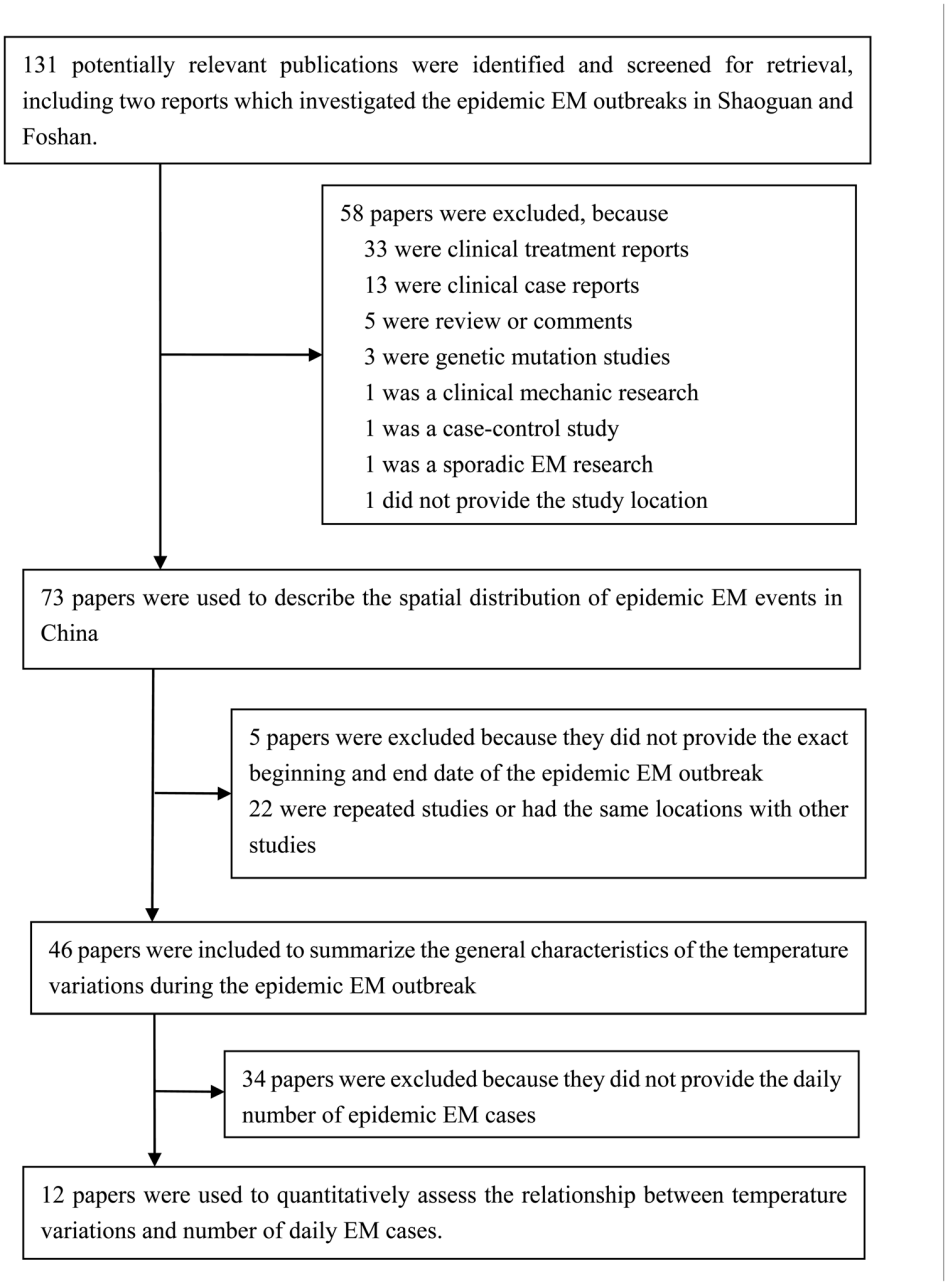
Flow chart of the study selection process.

Because there is no laboratory test that can verify the diagnosis of EM, papers that employed the following inclusion criteria (symptoms and signs) to define an EM case were included in the potential relevant papers pool: burning extremity pain, pain aggravated by warming, pain relieved by cooling, erythema of affected skin, and increased temperature of affected skin^41^. In order to demonstrate the spatial distribution of epidemic EM events in China, all the relevant papers which met the following criteria were included: (a) subjects must be primary epidemic EM cases; (b) time and location of study must be provided; and (c) the study location must be in China. Secondly, in order to summarize the temperature characteristics during an epidemic EM outbreak, papers that further met the additional following criteria were included: (d) must provide the exact beginning and end date of an epidemic EM outbreak. Finally, in order to assess the relationship between temperature fluctuation and number of daily EM cases, all the papers that met criteria (a) to (d), and also provided the daily number of epidemic EM cases were included. If multiple studies covering the same population were identified, the article with the most information was selected. Accordingly, papers that did not meet the above criteria were excluded. The process of study selection is presented in detail in Figure 1. In addition, we also included two reports that investigated the epidemic EM outbreaks in Shaoguan of 2009 and Foshan of 2014 in Guangdong province, China, respectively (See supplementary materials 1 and 2).

### Data extraction and collection

For each included paper which met criteria (a), (b) and (c), we extracted the following information: authors, year, source of publication, time and location of the epidemic EM outbreak. For studies which further met criteria (d), the exact date and prevalence of EM were added. For studies which met all the inclusion criteria, we also collected the number of daily EM cases and a range of daily temperature measures including mean temperature (TM), maximum temperature (Tmax), minimum temperature (Tmin), apparent TM, apparent Tmax, and apparent Tmin. If the temperature data were not provided in the papers, we obtained it from the China Meteorological Administration for the city where the outbreak occurred.

All data extraction was carried out by two authors using a standard form, and minor discrepancies were resolved by discussion. Apparent temperature indexes were calculated by the method provided by the Bureau of Meteorology, Australia (www.Bom.Gov.Au/info/thermal_stress).

### Statistical analysis

Firstly, ArcGis (ArcMap 9.3, Environmental Systems Research Institute, Redlands, USA) technology was employed to describe the spatial distribution of epidemic EM events from 1950 to 2014 in China.

In order to explore which temperature index (mean temperature, maximum temperature, minimum temperature, or their corresponding apparent temperature) was the best predictor of EM outbreaks, we used a two-stage model to analyze the data from the twelve papers which reported the number of daily EM cases. In the first stage, a multiple linear regression model was used to estimate the association between different temperature indicators and number of daily EM cases in each paper. A partial regression coefficent (β) for a one degree Celsius increment and its standard error (SE) were reported. The analysis duration was restricted to the time of temperature rising in each study, because most studies found that the majority of EM cases occurred during the rising of temperature. In the second stage, a meta-analysis model was employed to estimate the summary β of different temperature indexes. The heterogeneity of the included studies was assessed using Cochran's Q statistic. If the heterogeneity test was significant, a random-effects model was used to estimate the pooled association between temperature indicators and daily EM cases. Otherwise, the estimated results based on a fixed-effects model were reported. The results indicated that mean temperature (TM) had the largest β with number of daily EM cases (Supplementary Figure 3). Therefore, all the analyses reported were conducted using TM.

To summarize the temperature characteristics during epidemic EM outbreaks, the TM and date were standardized. We firstly defined the beginning and end date of each EM outbreak. Then, the TM for each day was subtracted by the Tmin during each EM outbreak. The Tmin was defined as the lowest daily TMs during an EM outbreak days. The day with the Tmin was defined as “0”, the date before the “0” was defined as minus, and the date after the “0” was defined as plus. For example, if the date of March 3^rd^ was defined as the “0”, March 1^st^ and March 5^th^ would be defined as “−2” and “+2” day, respectively. Then we calculated the average standardized TM in each standardized date for all included papers. A line graph was used to reveal the fluctuation of standardized TM. In order to qualitatively assess the association between TM fluctuation and incidence rate of EM, we divided all 24 papers which reported the incidence rate of EM into three groups: group 1 (the incidence rates of EM were less than 10%), group 2 (the incidence rates of EM were between 10% and 20%) and group 3 (the incidence rates of EM were larger than 20%). The line graph of TM fluctuation *vs* date was respectively made for each group.

Finally, a multiple-line graph was used to explore the relationship between TM and number of daily EM cases in the twelve papers, and the summary association was also reported based on the above two-stage model. In addition, we also conducted a sensitivity analysis to test the robustness of our findings, in which the study with the largest β, smallest β and both were respectively removed.

All statistical tests were two-tailed, and p < 0.05 was considered statistically significant. We used R software (version 2.15.2; R Development Core Team 2012, www.R-project.org/) to analyze the data. The “Metafor” package was used to fit meta-analysis.

## Results

### Search results

A total of 73 papers reported outbreaks of epidemic EM in China and provided the study location. Of them, 46 papers provided the exact beginning and end date of epidemic EM outbreak, in which, 12 papers further provided the number of daily epidemic EM cases.

### Epidemiological characteristics of epidemic EM

**Figure 2:**
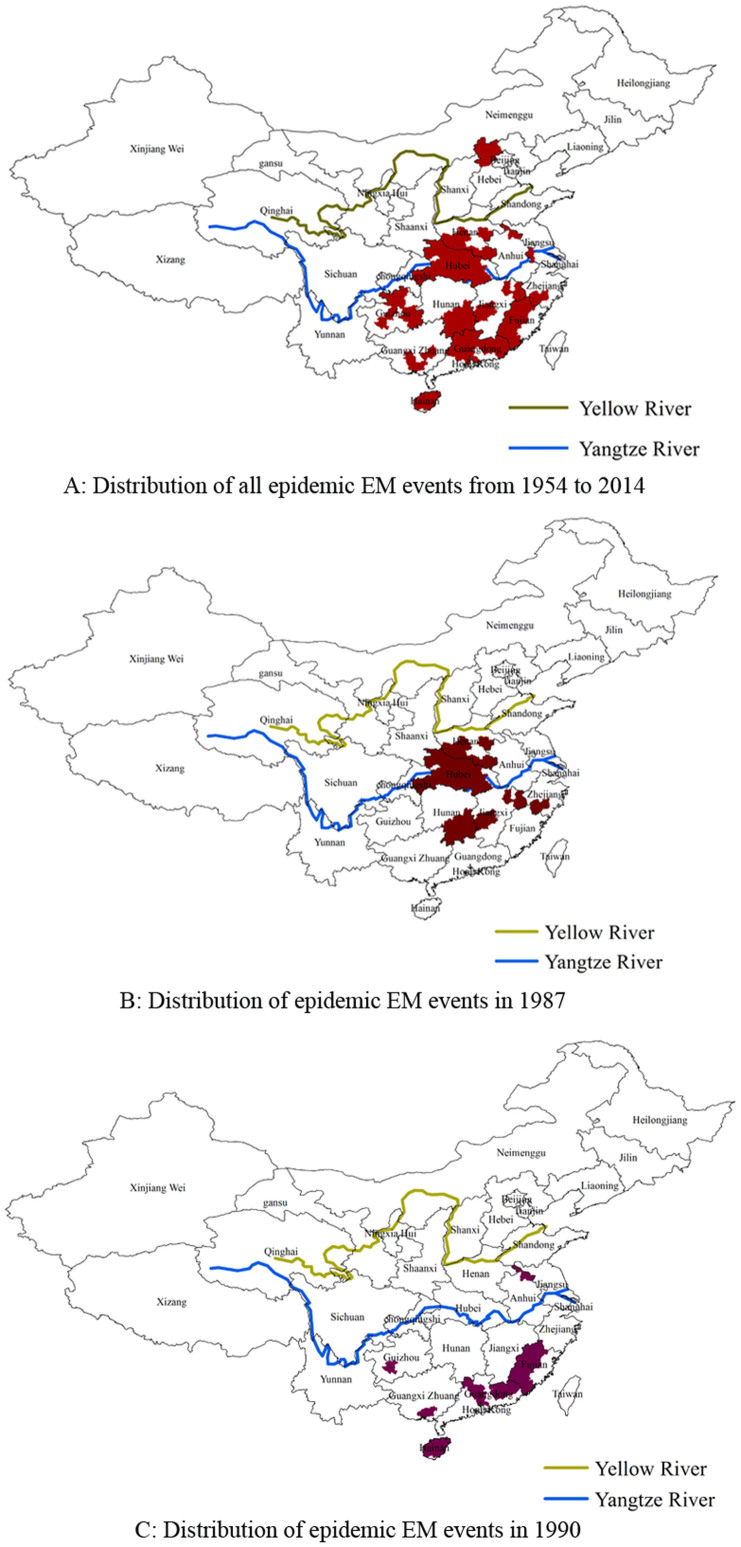
The distribution of epidemic EM events in China. *Note*: These maps were generated by ArcMap 9.3 software (Environmental Systems Research Institute, Redlands, USA).

A total of 54 regions located in 13 provinces experienced epidemic EM outbreaks from 1960 to 2014. Most regions were located in the southern part of China (south of the Yellow River). Two large outbreaks occurred in 1987 and 1990. The 1987 outbreak happened in central provinces in China including Hubei, Hunan and Jiangxi provinces (near the middle part of the Yangtze River), while the 1990 outbreak was mainly in south China including Fujian, Guangdong and Hainan provinces (Figure 2).

Almost all the epidemic EM events happened between February and March when ambient temperature experienced a large fluctuation. The EM cases included students, young labor workers, young medical workers and soldiers, but the majority were middle school students. Furthermore, the incidence rates of EM were higher for female students (22.6%) than male students (9.5%), and for resident students (24.5%) than non-resident students (9.4%).

### Clinical characteristics of epidemic EM

The major clinical characteristics of EM cases included burning, sharp, tingling and/or stinging pain in toes, soles and/or dorsum of feet, increased temperature, erythema and swelling. Some cases reported burning pain in fingers, wrists and knees. These symptoms were more severe during the night, walking, standing, exercising and when the individual was near a heater. Patients could relieve their pains by rest, elevating feet, using cold packs, walking on cold floors, immersing feet in cold water, or sleeping with the affected part outside the quilts. Some patients used an electric fan to keep cooling their feet. These symptoms were reported as both sustained or paroxysmal. The duration of symptoms varied among cases with most cases lasting from minutes to hours, but in a few patients the symptoms continued for one to two days, or even years.

### The characteristics of temperature during epidemic EM outbreaks

**Figure 3:**
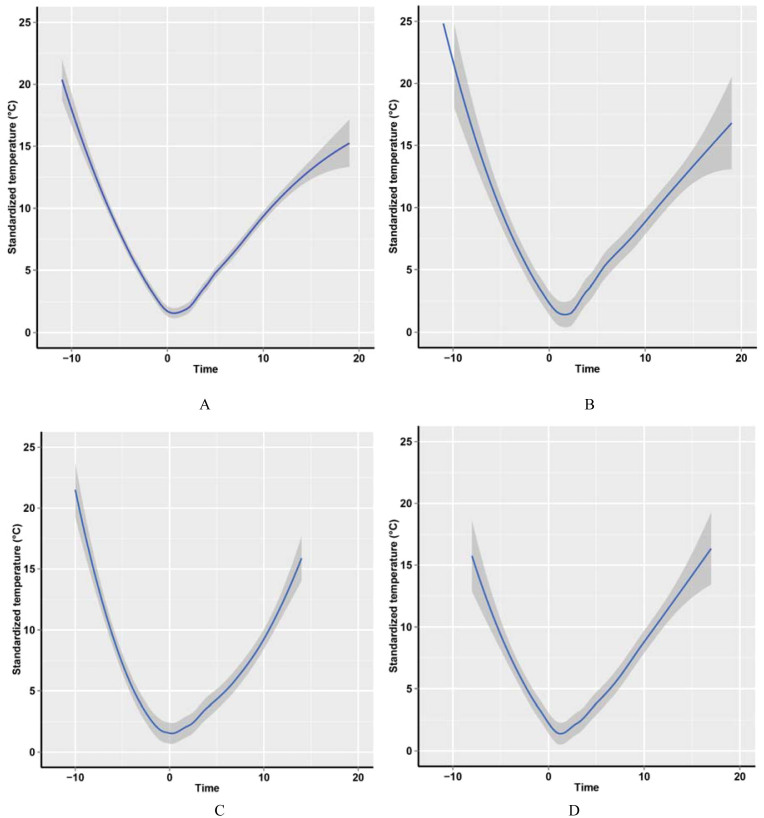
The standardized temperature during epidemic EM events. Note: The standardized temperature was defined as the average temperature subtracted by the minimum temperature during each EM outbreak. The day with the minimum temperature was defined as “0” in the time axis, the date before the “0” was defined as minus, and the date after the “0” was defined as plus. (A): Relationship between the temperature and time in all included articles. (B): Relationship between the temperature and time in articles with the incidence of EM less than 10.0%. (C): Relationship between the temperature and time in articles with the incidence of EM between 10.0% and 20.0%. (D): Relationship between the temperature and time in articles with the incidence of EM larger than 20.0%.

A “V”-shaped fluctuation of TM during epidemic EM outbreaks was observed in data obtained from 46 studies (Figure 3). The characteristics of the fluctuation were that firstly, the TM sharply and continuously dropped to a low level, usually lower than the minimum temperature during the same periods in neighboring years. The mean duration and decline rate of TM were 6.9 days and 2.2°C/day, respectively. Subsequently, TM rapidly increased to a high level. The mean duration and increment rate of TM for the entire epidemic event period were 12.5 days and 1.2°C/day respectively (Table 1). Most epidemic EM cases occurred during the TM increase period. After the TM became stable at a higher level, nil or very few EM cases were reported.

The 24 studies which reported the incidence rate of EM were divided into three groups, and the number of studies were 10, 6 and 8, respectively. Compared with group 1, TM after time “0” rose slightly faster in group 2 and group 3 (Figure 3).

### Associations between TM fluctuation and number of daily EM cases

Table 2 shows the characteristics of the 12 studies selected to analyze the associations between TM fluctuation and EM cases. The event occurred between February and March in all studies. These studies came from 6 provinces of southern China, and included a total of 3,874 EM cases. Almost all cases in these studies were middle school students, except for one study which did not provide the details on occupation.

**Figure 4:**
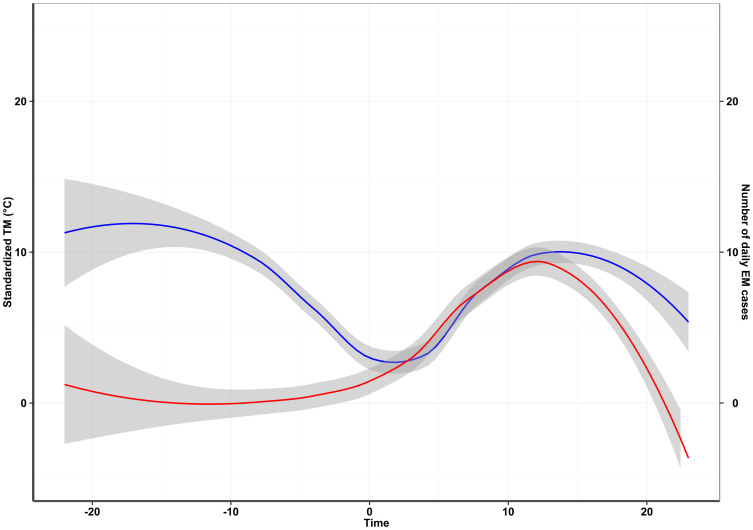
The relationship between standardized temperature and number of daily EM cases. Note: The standardized temperature was defined as the average temperature subtracted by the minimum temperature during each EM outbreak. The day with the minimum temperature was defined as “0” in the time axis, the date before the “0” was defined as minus, and the date after the “origin” was defined as plus. The red line indicates the number of daily EM cases, and blue line indicates the standardized TM.

**Figure 5:**
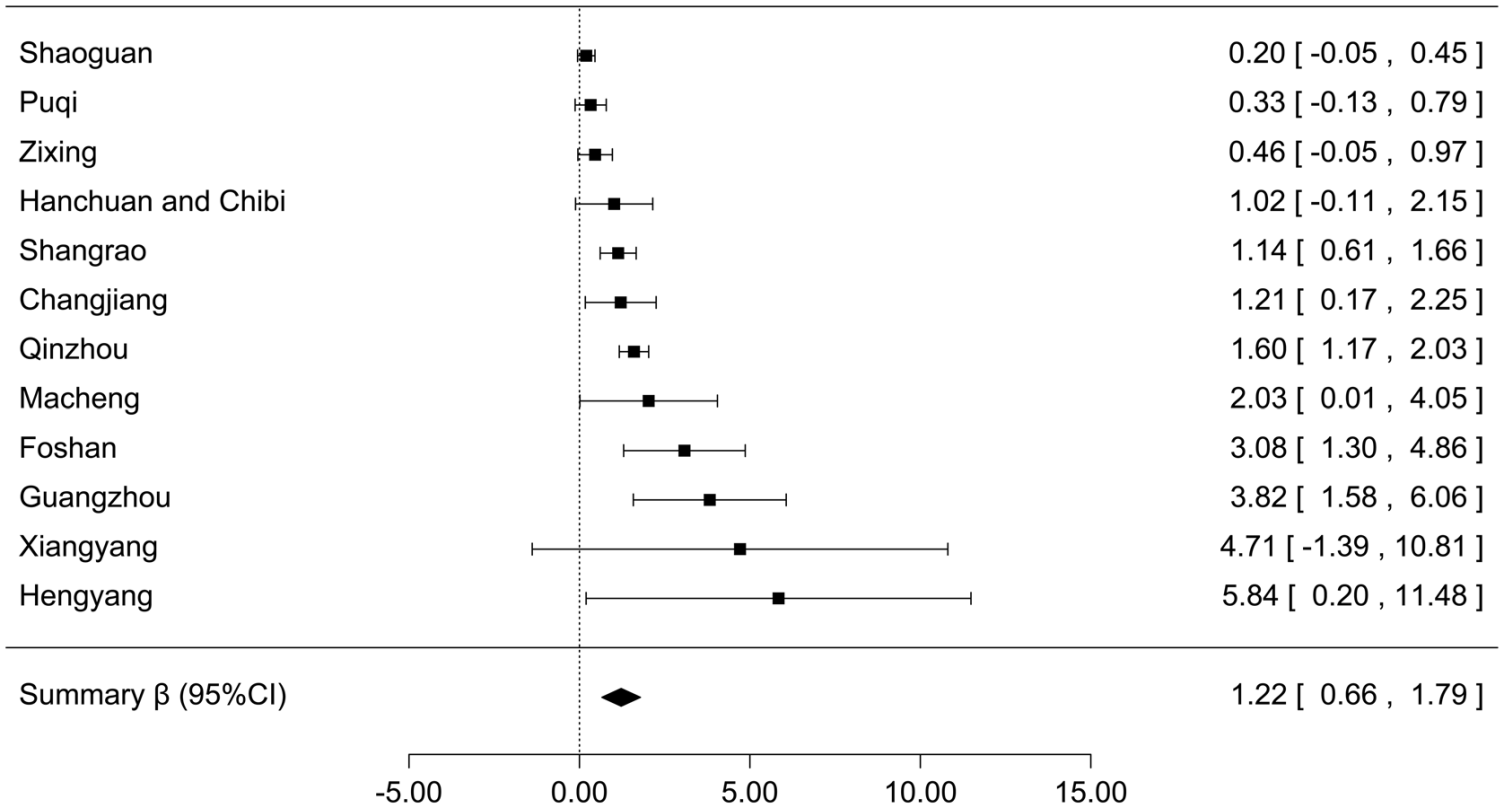
Forest plot of the summary association between daily average temperature and number of daily EM cases.

Figure 4 shows the relationship between standardized TM and the number of daily EM cases in the 12 studies. We observed that TM had a similar fluctuation with the daily number of EM cases after day “0”. A similar graph was drawn for each included study (Supplementary Figure 4). The results of meta-analysis show that the summary β of TM was 1.22 (95%CI: 0.66 ~ 1.79), which indicated that one degree Celsius increment was associated with an average increment of 1.22 cases (Figure 5).

### Sensitivity analysis

**Figure 6:**
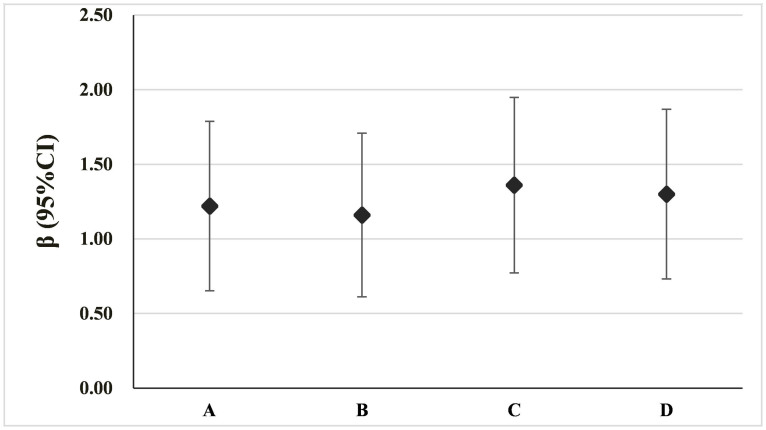
Sensitivity analyses of meta-analysis methods on the association between daily average temperature and number of daily EM cases. (A): Summary β was estimated in all included papers. (B): Summary β was estimated by removing the largest β. (D): Summary β was estimated by removing the smallest β. (D): Summary β was estimated by simultaneously removing the largest and smallest βs.

Sensitivity analyses indicate that the results were robust to the estimation methods and to removing any single study (Figure 6).

## Discussion

Although erythromelalgia epidemics have been quite common in China, few studies from China have been reported in the international literature^1^. This study collected epidemic EM data from previously published papers in China to conduct a comprehensive analysis. We found that epidemic EM outbreaks were not rare in China, especially in southern China, and the “V”-shaped fluctuation of TM between February and March was significantly associated with an epidemic EM outbreak.

The spatial distribution of epidemic EM cases show that most EM outbreaks occurred in southern China. This interesting spatial distribution may be related to the local climatic characteristics and inadequate adaptive capacity. During the late winter and early spring (February and March), temperature usually has a large fluctuation in China. For example, during mid-February in 1987, under the influence of El Nino, three episodes of cold air mass from Siberia moved into central China, which led to a sharp decline in temperature. The minimum temperature in some regions of Hubei province was 2°C lower than the average level in the same period of the past 34 years, and temperatures dramatically declined by 17.8°C within 48 hours. This was closely followed by a warm air mass from the Pacific Ocean that caused the consequent rapid rising of temperature in this region^38^. This large fluctuation of temperature might cause thermoregulatory, automatic nerve and peripheral vascular contraction dysfunction, and hence induced epidemic EM^11^,^14^,^20^. In northern China, the heating system could mitigate the effect of this temperature fluctuation on human health. However, in southern China, where cold weather is not common, heating systems are not common and this contributes to higher exposure to the varied ambient temperature^42^. In addition, populations in southern China have acclimatized well to the warm weather but it is possible that when exposed to the cold air mass, the rapid decline in temperature may exceed the range of their adaptive ability, and potentially induce the thermoregulatory dysfunction^20^.

The majority of EM cases in this study were middle school students, particularly female and resident students. We suggest some possible reasons for this. Firstly, these students are in an age of rapid development, and their automatic nervous and thermoregulatory systems are likely to be more sensitive to a large temperature fluctuation during a short period. In addition, they, especially resident students, often participate in less outdoor activities, have less emotional care from their families, poor accommodation and nutrition supply, factors which also increase their sensitivity to ambient temperature impacts^43^. However, it is unclear why female students were more vulnerable to EM. Some studies have postulated that it may be related to the relatively lower physical activities compared with males and endocrine hormone changes^44^. Research conducted in the future should explore the exact mechanisms of these differences between the sexes, such as mechanisms concerning immunity or heritage.

As there is no laboratory test that can verify the diagnosis of EM, all the previous cases were diagnosed based on clinical characteristics and symptoms^3^. This may lead to misclassification of other common diseases, such as chilblain. Chilblain is a localized disease which presents as inflammatory, erythematous or purple, intensely pruritic or painful acral lesions^45^. It is commonly seen in susceptible individuals after prolonged exposure to nonfreezing cold temperatures and damp conditions^46^. This disease is commonly seen in Chinese people, especially in young students from rural areas^47^. Although it has similar symptoms with EM, some remarkable symptoms, such as intensely pruritic and inflammatory that often attack the face, ears and dorsal side of hands and feet^47^, distinguish it easily from EM. More importantly, most chilblain cases occur during the rapid decline of ambient temperature, and the peak of cases usually occurs near the lowest temperature^48^. By contrast, EM has some symptoms that are specific, such as pain aggravated by warming and relieved by cooling and in contrast EM usually occur during the rapid rising phase of ambient temperature.

We observed a common “V”-shaped fluctuation of TM during the epidemic EM outbreaks. We believe that this “V”-shaped fluctuation of temperature probably trigger the epidemic EM outbreak. During a sharp decline of temperature, it is likely that the acral small superficial arteries intensely constrict and dilate^3^, which induces the enhanced responses of these arteries to temperature. During the following rapid increase of temperature, the intense expansion of capillaries irritate the nerve endings around, and thus lead to syndromes including burning pain, increased temperature, erythema and swelling^11^,^14^,^20^. In addition, we further quantitatively assessed the relationship between EM cases and different temperature indicators, and found that TM, compared with Tmax, Tmin, and their apparent temperatures, had the most pronounced association with EM cases. Some previous studies have also demonstrated that TM is a better predictor of mortality and morbidity fluctuation than Tmax and Tmin^49^, since TM represents an average exposure based on the whole day while Tmax and Tmin only reflect the exposure for a short period. These findings demonstrate that the pattern of ambient temperature fluctuation might be a pivotal predictor of epidemic EM, and provide significant information on building up early warning system of epidemic EM especially for vulnerable populations like middle school female students.

Some limitations should be considered when interpreting our results. Firstly, the prevalence of epidemic EM may be underestimated in China because this syndrome is not monitored in routine disease surveillance system, and few clinical doctors are aware of the diagnosis and treatment of EM. This may cause selection bias. Secondly, publication bias can not be avoided because most data in this study was obtained from the published papers. Thirdly, not all included papers reported the incidence rates of epidemic EM, which may induce information bias on the total estimated incidence of EM. Fourthly, due to the large variation of study periods and locations, we can not easily determine the threshold of temperature which triggered the outbreak of epidemic EM. Lastly, no study has reported epidemic EM outbreaks in other countries. It is not clear why this syndrome is specific in China, and why it has association with climatic factors. More research should be conducted to fill these knowledge gaps.

## Conclusions

Epidemic EM appears quite common in southern China. Middle school students, especially female and resident students appear to be the most vulnerable groups. The “V”-shaped fluctuation of daily average temperature during the late winter and early spring might trigger an epidemic EM outbreak. As climate change proceeds, more EM outbreaks may occur because extreme weather events are projected to increase in coming decades. We suggest that epidemic EM should be included in the current routine disease surveillance system, and establish an early warning system of epidemic EM based on measured or projected temperature fluctuation in China.

## Author Contributions

T.L., Y.Z., X.L., H.L. and W.M. designed the study. T.L., X.L., J.X., W.Z. and Y.G. did the literature search and data collection. T.L. and X.L. managed and analyzed the data. T.L., S.T., S.R. and W.M. conducted the quality control, post-processing and oversight. All authors discussed and commented on the manuscript.

## Supplementary Material

Supplementary Information Supplementary material

## Figures and Tables

**Table 1:** Characteristics of daily mean temperature fluctuation during the epidemic EM outbreaks in the 46 included papers

	Mean	Minimum	25th	75th	Maximum
Temperature decline phase					
Duration of decline (days)	6.9	3	5	9.3	13
Decline rate (°C/day)	2.2	1.1	1.7	2.7	4.7
Temperature increase phase					
Duration of increase (days)	12.5	4.0	9.0	15.0	27.0
Increase tate (°C/day)	1.2	0.5	0.9	1.4	2.1

**Table 2:** General characteristics of studies that reported the number of daily EM cases and temperature in China

Reference	Subjects	Location	Time of epidemic erythromelalgia event	Number of cases
Liang et al (1989)	Middle school students	Qinzhou, Guangxi	March 10^th^–17^th^, 1988	116
Luo et al (1987)	Middle school students	Shangrao, Jiangxi	February 19^th^–March 11st, 1987	296
Wang et al (1985)	NA	Guangzhou, Guangdong	February 28^th^–March 15^th^, 1959	407
Mo et al (1987)	Middle school students	Hanchuan and Chibi, Hubei	February 10^th^–March 10^th^, 1987	248
Wang et al (1992)	Majority were middle school students	Changjiang, Hainan	February 25^th^–March 13^rd^, 1987	147
Liu et al (1989)	Middle school students	Xiangyang, Hubei	February 26^th^–March 6^th^, 1987	417
Li et al (2011)	Middle school students	Zixing, Hunan	March 1^st^–28^th^, 2010	154
Shi et al (1989)	Middle school students	Macheng, Hubei	February 10^th^–March 10^th^, 1987	499
Xu et al (1987)	Middle school students	Puqi, Hubei	February 10^th^–March 12^nd^, 1987	78
Dong et al (1987)	Middle school students	Hengyang, Hunan	February 27^th^–March 6^th^, 1987	910
GDCDC (2009)	Middle school students	Shaoguan, Guangdong	February 15^th^–March 24^th^, 2009	108
GDCDC (2014)	Middle school students	Foshan, Guangdong	February 11^th^–March 3^rd^, 2014	494

NA: not available.

GDCDC: Guangdong Provincial Center for Disease Control and Prevention.
