# Supplementary material for: A large temperature fluctuation may trigger an epidemic erythromelalgia outbreak in China
Source: Sci Rep. 2015 Mar 30;5:9525. doi: 10.1038/srep09525 (PMC4377627; doi:10.1038/srep09525)
Supplement: Supplementary Information — Supplementary material [file srep09525-s1.pdf]

A large temperature fluctuation may trigger an epidemic erythromelalgia outbreak in China

Tao Liu, Yonghui Zhang, Hualiang Lin, Xiaojuan Lv, Jianpeng Xiao, Weilin Zeng, Yuzhou Gu, Shannon

Rutherford, Shilu Tong, Wenjun Ma

Supplementary material 1: The epidemic EM outbreak in Shaoguan of 2009

On the March 24th of 2009, Shaoguan Center for Disease Control and Prevention reported many students from several local middle schools experienced severe pain in extremities with unknown reasons. Through the field investigation, a total of 108 cases were diagnosed as EM. In order to understand the detailed epidemiological and clinical characteristics of the EM cases, an investigation was conducted in one local high school. Fifty-six EM cases were found, with an incident of 6.6%. The first case was found on the February 15th, the second case was reported on March 8th, and the onset peak period was during the March 15th-18th (Supplementary Figure 1). Of the total cases, 51 (91.1%) were female students, and 53 (94.6%) were resident students. They also had common clinic characteristics including burning pain in toes (83.9%), burning pain in soles (57.1%), tingling in the feet (35.7%), and feeling hot in the feet (0.4%).

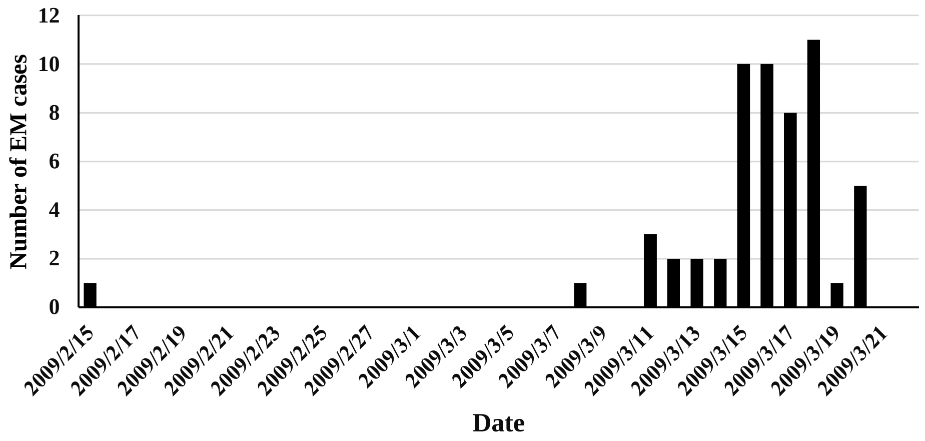

Supplementary Figure 1. The temporal distribution of EM cases in Shaoguan of 2009

Supplementary material 2:The epidemic EM outbreak in Foshan of 2014

On the February 27th of 2014, Foshan Center for Disease Control and Prevention reported an outbreak of unknown reason pain in extremities in several local middle schools. The common clinic characteristics were burning pain in toes and/or soles. A field investigation was conducted, and found a total of 494 EM cases. A detailed investigation was conducted in a high school to understand the epidemiological and clinical characteristics of this EM outbreak. A total of 172 EM cases were found, with a prevalence of 12.1%. The first case was reported on February 14th. Two onset peak periods were observed on the February 20th and during the February 23rd-27th, respectively (Supplementary Figure 2). In the total EM cases, 152 (88.4%) were female students, and 145 (84.3%) were resident students. The cases with burning pain in toes, soles, heels and fever were 136 (79.1%), 120 (69.8%) 66 (38.4%) and 18 (10.5%), respectively.

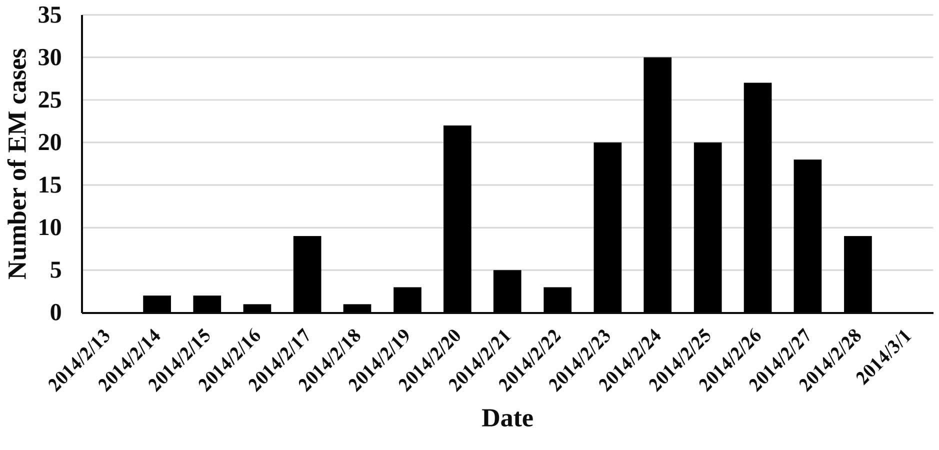

Supplementary Figure 2. The temporal distribution of EM cases in Foshan of 2014

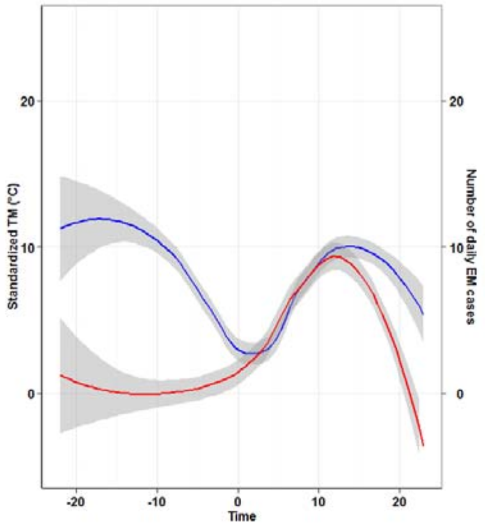
Standardized TM (°C)
Number of daily EM cases
Time
20
10
0
-20
-10
0
10
20

A

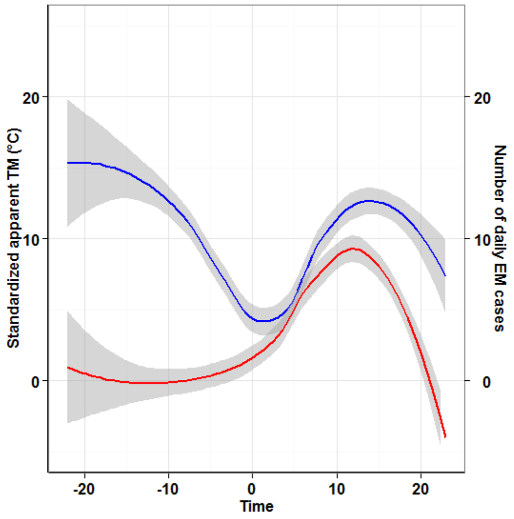
Standardized apparent TM (°C)
Number of daily EM cases
Time
20
10
0
-20
-10
0
10
20

B

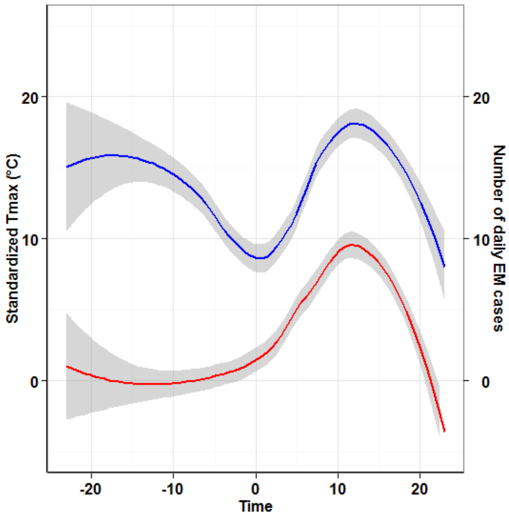
Standardized Tmax (°C)
Number of daily EM cases
Time
20
10
0
-20
-10
0
10
20

C

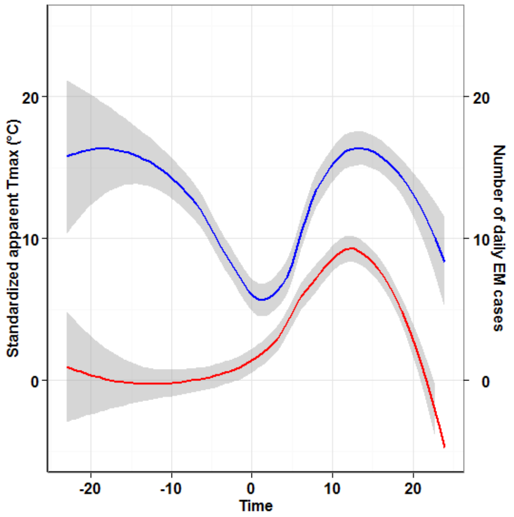
Standardized apparent Tmax (°C)
Number of daily EM cases
Time
20
10
0
-20
-10
0
10
20

D

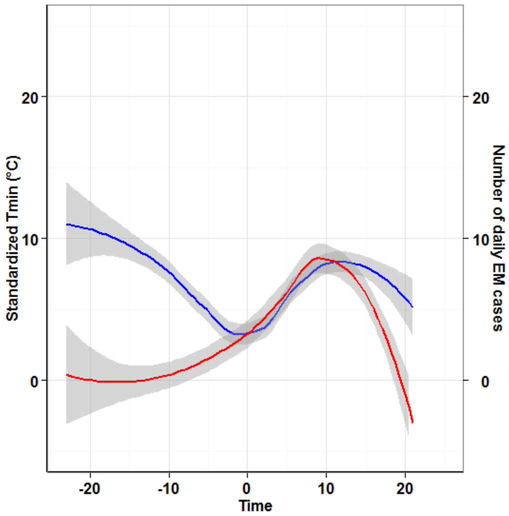
Standardized Tmin (°C)
Number of daily EM cases
Time
20
10
0
-20
-10
0
10
20

E

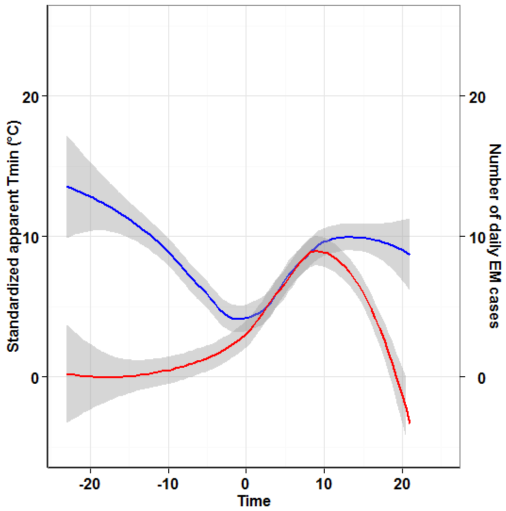
Standardized apparent Tmin (°C)
Number of daily EM cases
Time
20
10
0
-20
-10
0
10
20

F

Supplementary Figure 3. The relationship between different standardized temperature indexes and number of daily EM cases

Note: The data in these figures were extracted from the twelve papers which reported the number of daily EM cases.

A: The relationship between standardized TM and number of daily EM cases. The summary partial regression coefficient (β) was 1.22 (SE=0.29).

B: The relationship between standardized apparent TM and number of daily EM cases. The summary β was 1.06 (SE=0.26).

C: The relationship between standardized Tmax and number of daily EM cases. The summary β was 1.13 (SE=0.25).

D: The relationship between standardized apparent Tmax and number of daily EM cases. The summary β was 0.88 (SE=0.19).

E: The relationship between standardized Tmin and number of daily EM cases. The summary β was 0.77 (SE=0.35).

F: The relationship between standardized apparent Tmin and number of daily EM cases. The summary β was 0.66 (SE=0.29).

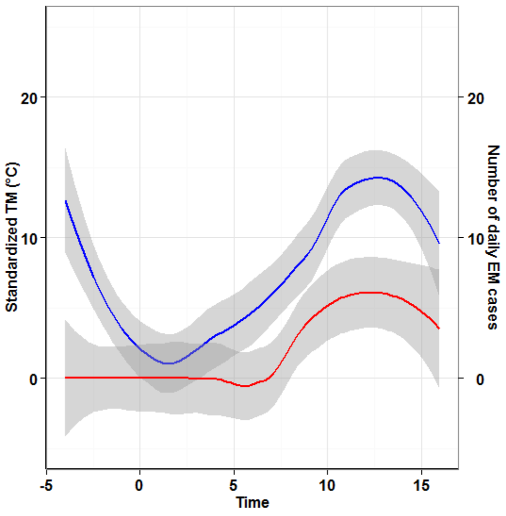

A: Qinzhou

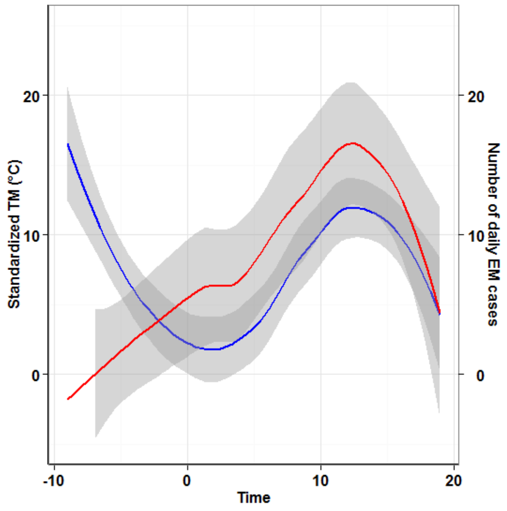

B: Shangrao

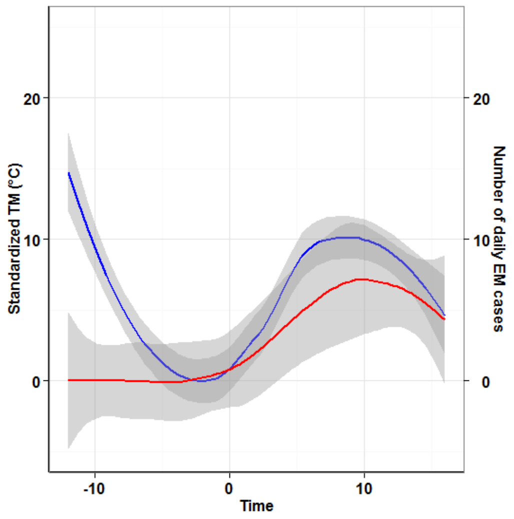

C: Guangzhou

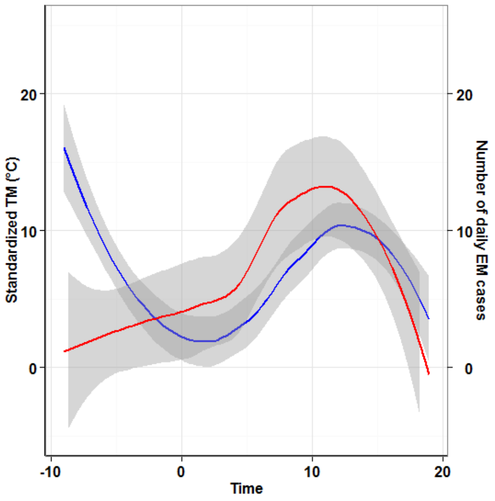

D: Hanchuan

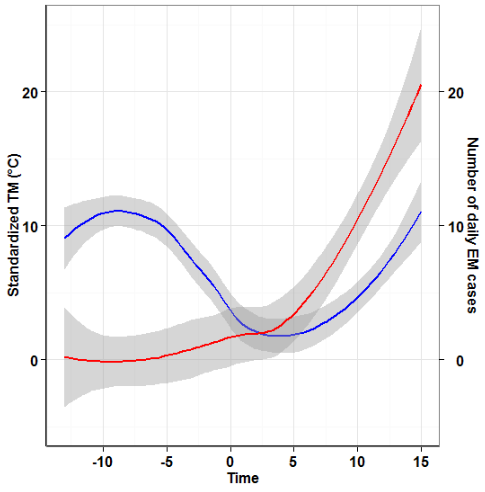

E: Changjiang

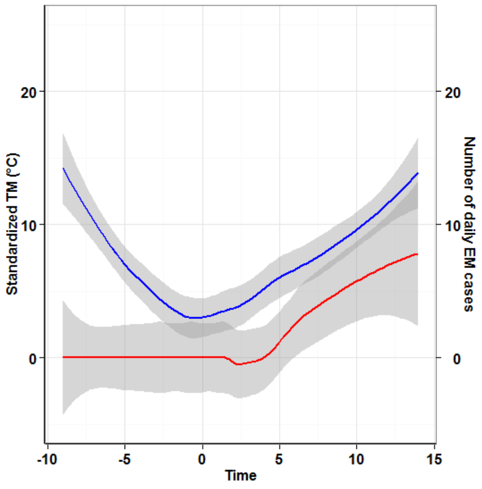

F: Xiangyang

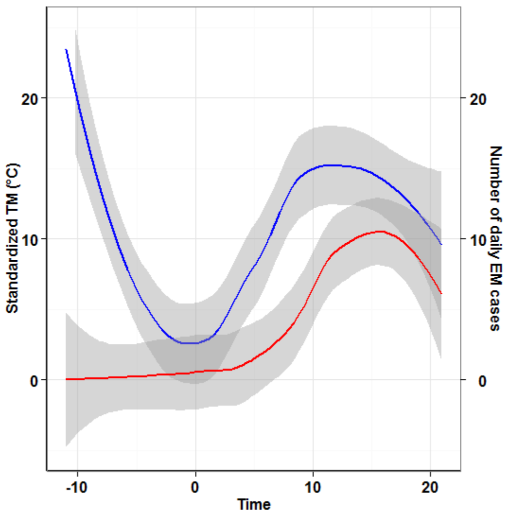

G: Zixing

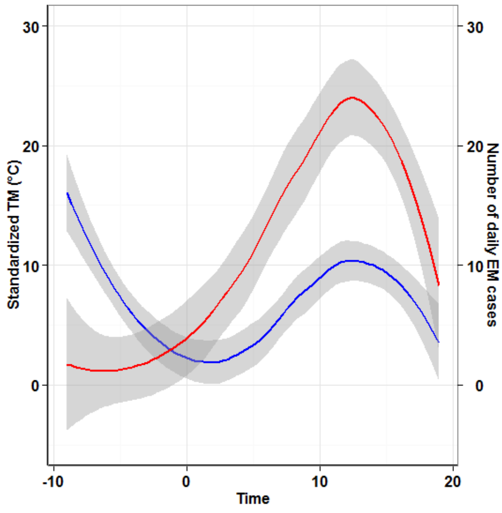

H: Macheng

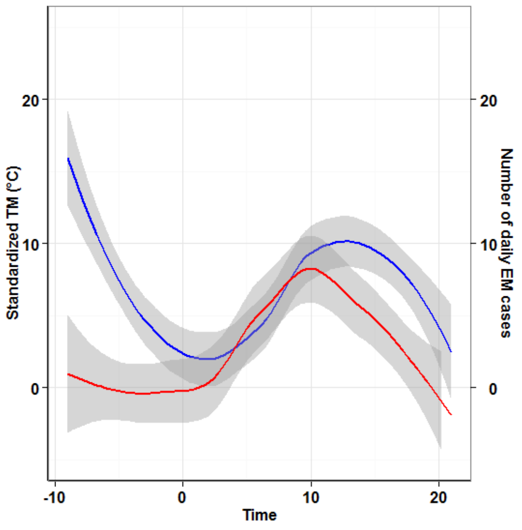

I: Puqi

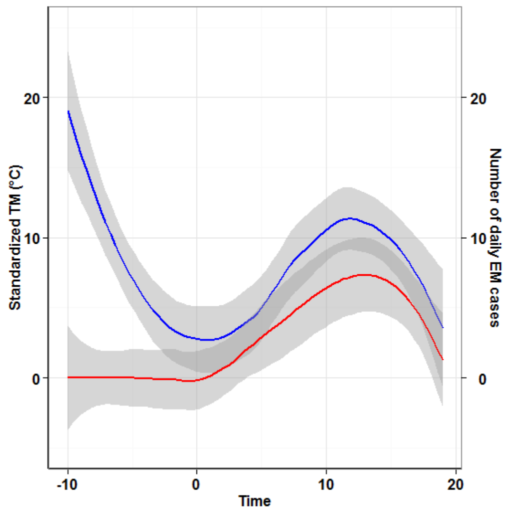

J: Hengyang

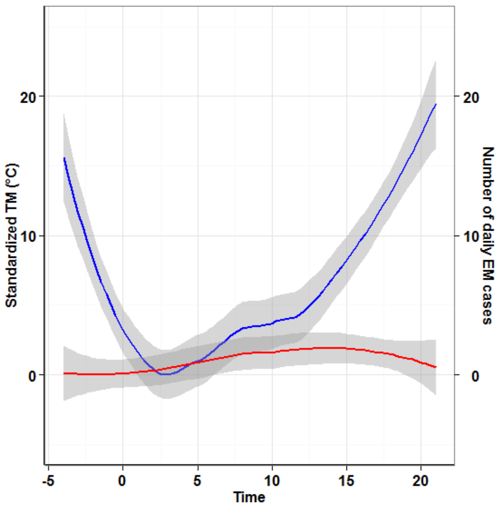

K: Shaoguan

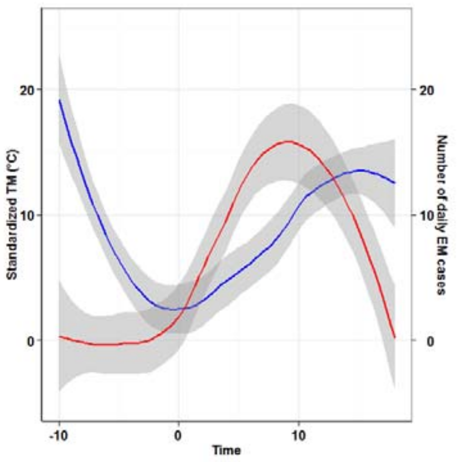

L: Foshan

Supplementary Figure 4. The relationship between standardized temperature and number of daily EM cases in each paper

Note: The standardized temperature was defined as the average temperature subtracted by the minimum temperature during each EM outbreak. The day with the minimum temperature was defined as “0” in the time axis, the date before the “0” was defined as minus, and the date after the “origin” was defined as plus.
